# Simulation of the Light Transmittance in Macroporous Silica

**DOI:** 10.3390/ma13071635

**Published:** 2020-04-01

**Authors:** Wenqi Zhu, Xingzhong Guo, Lan Wu, Hui Yang

**Affiliations:** 1 Zhejiang-California International Nanosystems Institute, Zhejiang University, Hangzhou 310058, China; wenqi_zhu@zju.edu.cn (W.Z.); wul@zju.edu.cn (L.W.); 2 School of Materials Science and Engineering, Zhejiang University, Hangzhou 310027, China; msewj01@zju.edu.cn; 3 Research Institute of Zhejiang University-Taizhou, Taizhou 318000, China

**Keywords:** macroporous silica, light transmittance, morphological parameters, finite element method

## Abstract

This paper focuses on the light transmittance of macroporous silica as a photocatalyst carrier. In addition to the characteristics of photocatalysts, the structure of porous bulk is also important since it affects the propagation of light. Realistic porous structures are generated by a Voronoi-based approach. Four morphological parameters are highly controlled during generating, that is, porosity, coefficient of variation, diameter ratio and normalized curvature. Finite element method (FEM) is used to simulate the propagation of light in the porous models in the visible light range. The intensity shows a quadratic decrease with the increase of the depth of light propagation. The influences of the morphological parameters on the light transmittance are analysed. It turns out that the porosity has a great influence on the light transmittance while the coefficient of variation and the diameter ratio have small ones. Moreover, the influence of the normalized curvature is little. Besides, the effect of the wavelength of visible light can not be ignored. With the simulation, the depth of visible light entering the porous silica can be estimated, which is challenging to access experimentally.

## 1. Introduction

Porous materials are widely used in numerous engineering fields due to their special pore structures and their excellent combination of multiple physical properties. The main application for polymeric and glass porous materials is as thermal insulation materials [1,2] owing to their low thermal conductivity, such as disposable coffee cups and the thermal-insulation equipments used in transport systems, modern buildings and booster rockets for the space shuttle. Thanks to their remarkable energy absorption [3,4,5] and vibration damping [6], metallic porous materials can be applied in the automotive industry. Hence over the years, people have paid much attention to the thermal properties [1,7,8,9,10,11,12] and the mechanical properties [4,5,13,14,15,16,17,18] of porous materials. Catalyst carrier [19], high-temperature applications [20,21], sandwich panels [22] and porous electrode for fuel cell [19] are also typical examples of porous material applications.

According to the characteristics of typical pollutants of indoor air, the porous adsorption has been supposed to combine with photocatalysis to purify indoor air [23]. The indoor air can be rapidly and efficiently treated due to the efficient adsorption of porous bulk materials and the visible light catalytic degradation of multi-metal composite oxides. Porous silica is an ideal photocatalyst carrier since it is structurally stable, chemically stable and inexpensive [24,25]. The recent literature mainly focuses on how to improve catalyst efficiency and how to prepare porous silica. Sol-gel TiO${}_{2}$ films have been used as photocatalyst to eliminate the microorganisms from indoor air in realistic conditions [26]. Reference [27] proves that the nano-silica addition and the ultraviolet pre-treatment can enhance the photocatalytic efficiency of the carbon-doped titanium dioxide coatings. Furthermore, the reaction rate and reactive uptake coefficient of the developed coatings have been computed. The relations between the characteristics of photocatalysts (e.g., surface area, surface chemistry and crystallinity) and photocatalytic activity have been investigated in Reference [28], as well as the deactivation of photocatalysts during photocatalytic oxidation processes and some regeneration techniques. Ag nanoparticles have been embedded into the porous silica uniformly to prepare photocatalyst by modifying porous skeleton [29]. After functionalized by an important variety of moities and techniques, silica monoliths with hierarchical porosity have been revealed to be a great opportunity for process intensification [30]. By combining the polymerization-induced phase separation with epoxide-mediated sol-gel process, hierarchically porous silica monoliths have been prepared with interconnected macropores and uniform spherical mesopores spontaneously [31]. With this method, the pore structures can be controlled precisely. Using wet impregnation method, polyethyleneimine has been modified on hierarchically porous silica monoliths to prepare a novel CO${}_{2}$ sorbent [23].

However, in order to improve the efficiency of such photocatalysis, it is not enough to consider only the characteristics of photocatalysts. Unlike other catalysis, photocatalysis requires the irradiation of light. Therefore, when the carrier is a porous bulk, how to make light propagate as deeply as possible in the complex porous structure is a very important problem. The deeper the light propagates, the more photocatalysts can be activated, thereby improving the efficiency of photocatalysis. Nevertheless, there is little literature on the relationship between porous structure and light propagation. It is not clear how to change the porous morphology and structure to facilitate light propagation.

In this work, the light transmittance of macroporous silica used in the field of photocatalysis is studied. In order to improve the efficiency of photocatalysis, it is necessary to pay attention not only to the characteristics of the photocatalyst, but also to the structure of the photocatalyst carrier, that is, macroporous silica in this paper, so that light can enter the carrier more effectively. With a methodology based on Voronoi diagram, the porous structures are numerically generated with a high control of several morphological parameters, which makes the structures realistic. The light transmittance of porous silica model is simulated by FEM. The numerical studies are performed to determine the representative element volume (RVE). The influences of the morphological parameters and the wavelength parameter are evaluated.

## 2. Generation of Porous Silica Models

**Figure 1:**
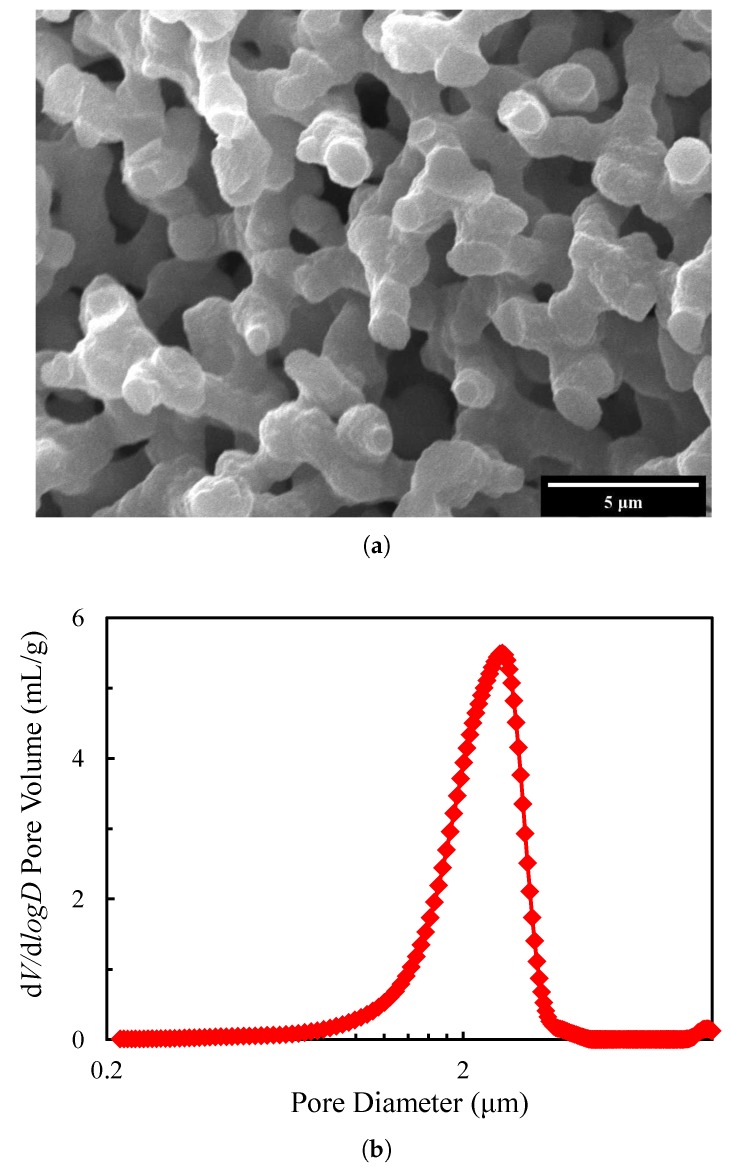
A typical porous silica structure: (**a**) observed through scanning electron microscopy (SEM); (**b**) pore size distribution.

The typical structure of porous silica prepared by sol-gel method can be observed in scanning electron microscope (SEM) images (Figure 1a). Mercury intrusion porosimetry [32] is used to evaluate the pore size distribution. As can be seen in Figure 1b, the pore size varies from 1.3 μm to 3.5 μm, and the distribution is concentrated. The median diameter is about 2.5 μm, which will be taken when generating the porous silica models.

Open-cell macroporous structures are numerically generated by the methodology based on Voronoi diagram. The methodology is summarized as follows:

- A certain amount of seeding points are created first, corresponding to the cells of the final structure. A Random Sequential Absorption Algorithm [33] is imposed to make the equal-sized spheres (centred at the created seeding points) distributed in the given space without overlapping for the random structures.
- The space is partitioned into polyhedral regions with the seeding points by Voronoi diagram [34].
- The resulting structure is optimized by Surface Evolver [35] for energy stability.
- Polygonal struts are created with the final skeleton.

Four morphological parameters are considered to represent the microstructure of porous models.

(1) Porosity, *p*, indicates the proportion of the gas phase. In this paper, the porosities of models are limited in the range $\left(65\%-85\%\right)$, corresponding to the products prepared by sol-gel method in experiments.
(2) Coefficient of variation, $C_{V}=\sigma_{d_{c}}/\bar{d_{c}}$, figures the dispersion of cell size distribution of random structures, where $\sigma_{d_{c}}$ and $\bar{d_{c}}$ are the standard deviation of cell diameter and the average equivalent cell diameter respectively. In this paper, the Gaussian unimodal distribution with different standard deviations is used for different cell size distributions.
(3) Diameter ratio, $t=d_{min}/d_{max}$, represents the ratio of the cross section diameter between the middle and the end of a strut, where $d_{min}$ and $d_{max}$ are the minimum and maximum diameter. Table 1 shows the illustration of the struts with different diameter ratios.
(4) Normalized curvature, $k=r_{c}/r_{s}$, governs the shape of the cross section of a strut, where $r_{c}$ stands for the curvature radius of the circumcircle of the cross section and $r_{s}$ is the curvature radius of the sides of the cross section. Table 2 exhibits the illustration of the cross sections with different normalized curvatures.

**Figure 2:**
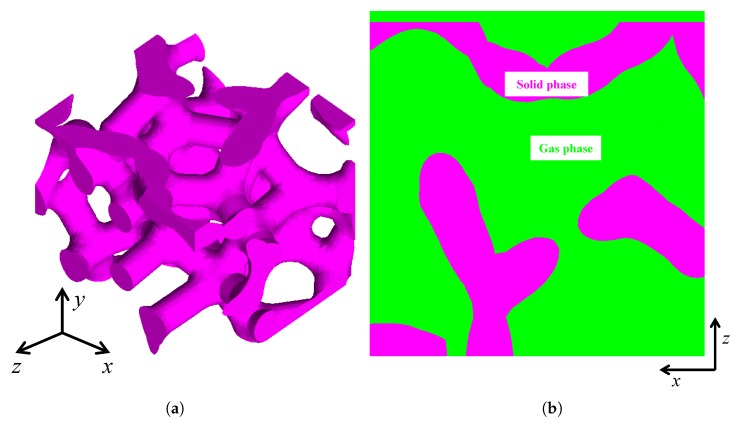
Illustration of (**a**) a porous structure and (**b**) a RVE composed of the solid and gas phases. (The reader is referred to the web version of this paper for interpretation of the references to color in this figure).

Using the above methodology realized by developing a Matlab code [36], a porous model with the domain dimension 3×3×3 (length × width × height) μm${}^{3}$ and the morphological parameters $\left\{p=75\%;C_{V}=0.1;t=1;k=1\right\}$ was generated as an example in the cubic frame (x,y,z) shown in Figure 2a. Since light propagates in both the solid and gas phases, the RVEs in this paper contain both phases, which are marked in purple and green respectively in Figure 2b from a two-dimensional perspective for clarity.

The RVEs with different morphological parameters were generated to study their effects in subsequent sections. Table 3 shows all the variables in the study and their values.

## 3. Numerical Simulation

**Figure 3:**
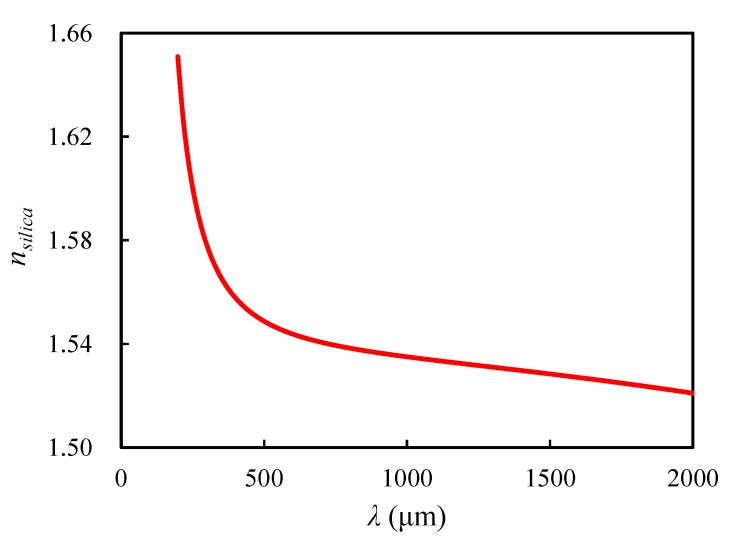
Evolution of $n_{silica}$ with respect to the wavelength.

The commercial package COMSOL Multiphysics 5.4 [37] was used for the numerical calculations. Since light is an electromagnetic wave, the electromagnetic wave and the frequency domain were selected as the physical field. The time-harmonic wave equation for the electric field was considered, that is,

(1) $$\nabla \times \left(\nabla \times E\right)-k_{0}^{2}\varepsilon_{r}E=0,$$

where E is the electric field phasor and $\varepsilon_{r}$ indicates the relative permittivity. The wave number of free space $k_{0}$ was defined as $k_{0}=\omega \sqrt{\varepsilon_{0}\mu_{0}}=\omega /c_{0}$, where ω is angular frequency, and $c_{0}=1/\sqrt{\varepsilon_{0}\mu_{0}}$ is the speed of light in vacuum. The refractive indexes of air and silica are built into COMSOL Multiphysics. For air, the real part and the imaginary part of the refractive index are $n_{air}=1$ and $k_{air}=0$, respectively. While for silica, the real and the imaginary parts are $n_{silica}$ and $k_{silica}=0$, respectively. Here, $n_{silica}$ is the function of the wavelength λ, and the evolution is shown in Figure 3.

**Figure 4:**
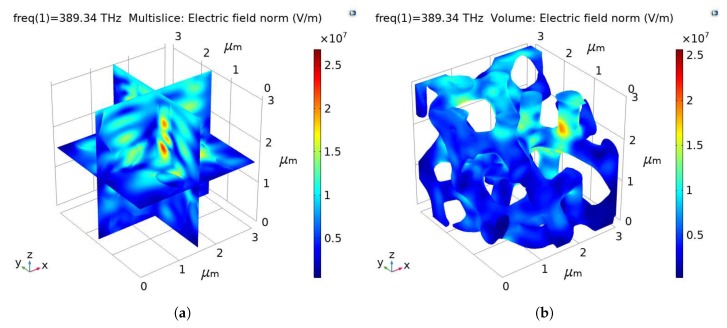
Illustration of the EFNof the sample RVE: (**a**) in the multislice perspective and (**b**) the EFN of the porous model.

The RVE generated in Section 2 was meshed with 405,042 linear tetrahedron solid elements using ICEM CFD 14.0 (ANSYS, Inc., Canonsburg, PA, USA) [38]. The scattering boundary condition was imposed on all boundaries. The port was selected as the upper surface of the RVE along *z* direction, and the direction of light propagation was defined as −z. The wavelength of visible light varies in the range (390–770) nm, and it was set to 770 nm in the study without special explanation. Figure 4 shows the electric field norm (EFN) (indicating the electric field intensity) of the sample RVE, where Figure 4a is in the multislice perspective and Figure 4b shows the EFN of the porous model.

**Figure 5:**
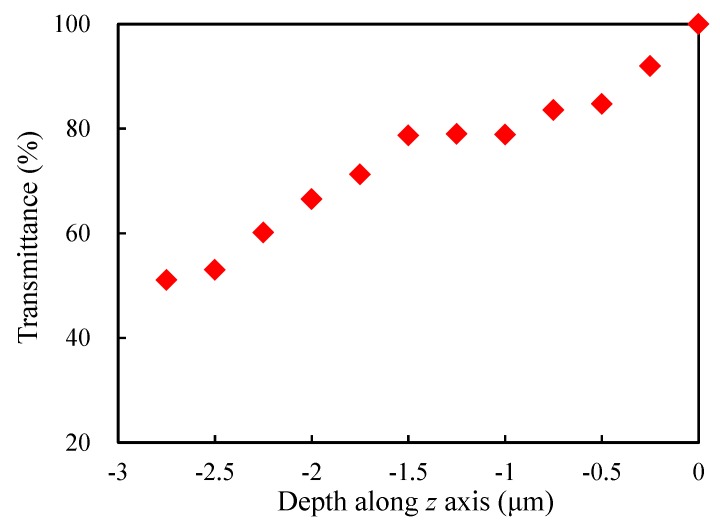
Evolution of the transmittance of the sample RVE with the depth along *z* axis.

Assuming that the *z* coordinate of the upper surface of the porous modelis equal to 0, since light propagates in the negative *z* direction, the transmittance was defined as the ratio of the average EFN in the xy-plane when *z* takes different values and that when *z* takes 0 (i.e., the average EFN of the upper surface). Figure 5 shows the evolution of the transmittance of the sample RVE with the depth along *z* axis. As can be seen from the figure, the light transmittance tends to decrease along the direction of light propagation but not monotonously, due to the random structure.

### 3.1. Mesh Sensitivity

**Figure 6:**
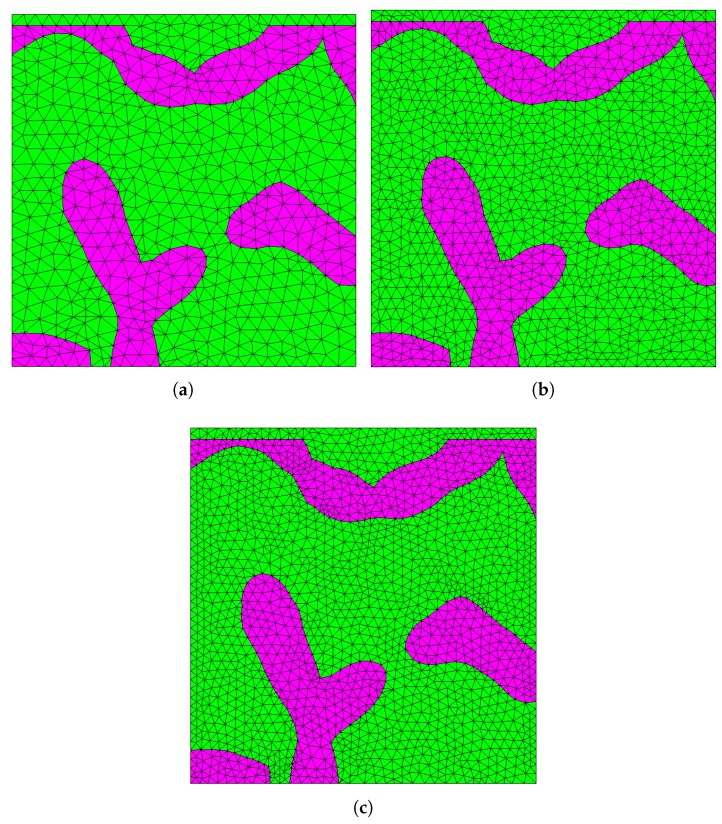
Illustration of the RVE meshed with (**a**) 173,769 elements, (**b**) 405,042 elements and (**c**) 750,416 elements.

In order to test the mesh sensitivity, the sample RVE was respectively meshed with 81,590 elements, 116,730 elements, 173,769 elements, 301,841 elements, 405,042 elements and 750,416 elements, which correspond to the global element seed size of 0.18 μm, 0.16 μm, 0.13 μm, 0.11 μm, 0.10 μm, 0.08 μm, respectively. Figure 6 shows the illustration of the RVE meshed with three different numbers of elements from two-dimensional perspective for clarity.

**Figure 7:**
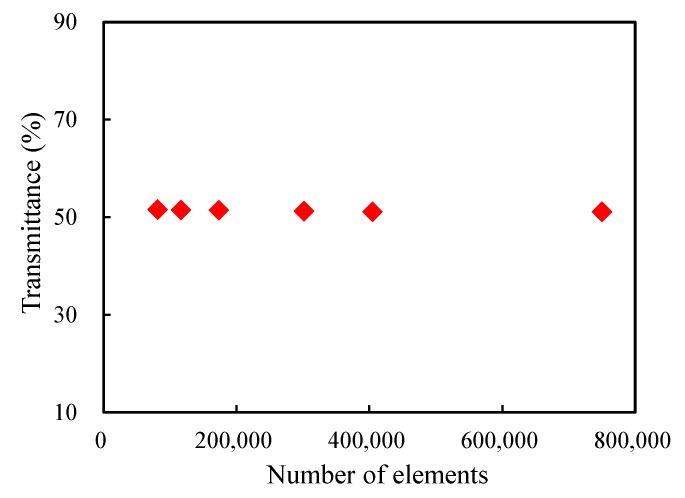
Evolution of the transmittance in the xy-plane with the number of elements for z=−2.75μm.

The transmittance in the xy-plane when *z* takes −2.75 μm was taken into account to study the influence of the number of elements. From Figure 7, one can see the transmittance has no significant variation with the change of the number of elements. This may be caused by the small difference in refractive index between air and silica, which reduces the effect of the mesh difference. While for COMSOL Multiphysics, there is a maximum limit on the size of the global element seed according to the wavelength, hence the global element seed size was chosen to be 0.10 μm without special instruction.

### 3.2. Boundary Value Problem

In order to study the boundary value problem, the height of the porous models was kept as 3 μm (in the *z* direction), and the length and the width are varied as 4×4 μm${}^{2}$, 5×5 μm${}^{2}$, 6×6 μm${}^{2}$, 7×7 μm${}^{2}$, 8×8 μm${}^{2}$, 9×9 μm${}^{2}$ and 10×10 μm${}^{2}$, respectively (considered as 7 different sets). Since the structures are random, for each set of models, numerous structures were generated and the statistical average value was calculated as the result. Furthermore, the number of realizations was determined by the convergence criterion [16,39], that is,

(2) $$\delta =\frac{|{\bar{T}}_{n}-{\bar{T}}_{n-1}|}{{\bar{T}}_{n-1}}⩽5\times 10^{-4}.$$

Here *n* denotes the number of realizations, and ${\bar{T}}_{n}$ indicates the statistical average value after *n* realizations. When the convergence criterion is satisfied, the obtained statistical average value can represent the final result of this set of models. In the following studies, all the results are the statistical average values.

**Figure 8:**
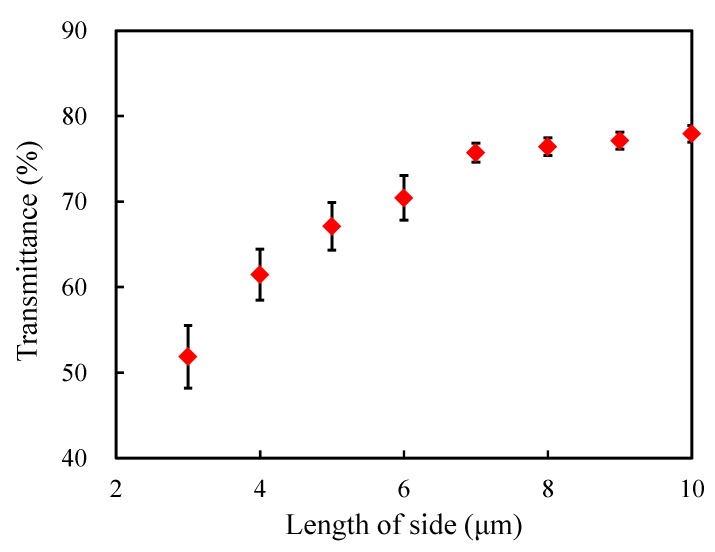
Evolution of the transmittance with the length of side.

Still considering the transmittance in the xy-plane when z=−2.75 μm, Figure 8 presents the influence of the model boundary on the transmittance. In the figure, the bars indicate the standard deviation errors of the statistical average values after certain numbers of realizations. The transmittance is around 50% when the model is small, and it increases with the increase of the length of side. Moreover, it tends to converge around 76% when the length of side reaches 7 μm. Hence the length and the width of porous models are set as 7×7 μm${}^{2}$ in the following studies.

## 4. Results and Discussion

### 4.1. Analysis of Light Propagation

**Figure 9:**
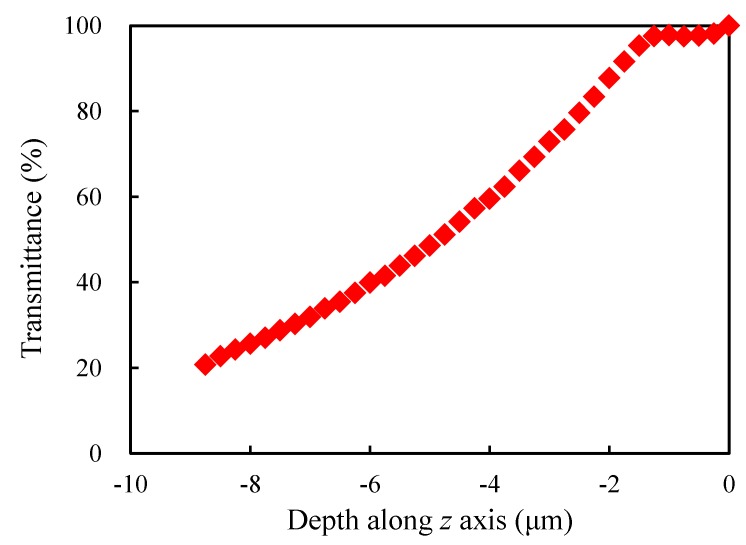
Evolution of the transmittance with the depth along *z* axis for the 7×7×9 μm${}^{3}$ models.

Numerous models were generated with the domain dimension 7×7×9 μm${}^{3}$ and the morphological parameters $\left\{p=75\%;C_{V}=0.1;t=1;k=1\right\}$ (thesame as those of the sample model). The statistical average value was considered for each xy-plane transmittance when *z* takes different values. For this set of models, the evolution of the transmittance with the depth along *z* axis is shown in Figure 9. From the figure, when light just enters the porous model, due to the complex porous structure, optical behaviors such as scattering and reflection make a large part of light stay in the surface layer. Simultaneously, the loss is relatively small, so there is no obvious variation in EFN.With the increase of the depth, losses such as absorption gradually increase, and behaviors such as scattering and reflection also allow less and less light to continue to propagate along the depth direction, which makes the EFN decrease, presenting a quadratic variation. At a depth of z=−8.75 μm, the intensity is about 20% of the initial one. Considering the shallow depth of light propagation, it is very difficult to measure or verify the propagation depth through experiments. Porous silica, prepared by the sol-gel method and used as a photocatalyst carrier, is usually produced into blocks or rods. The porous silica has a certain strength in the original state, while it becomes easily broken when cutting and grinding it into very thin slices to measure the transmittance at different thickness. In addition, samples of this thickness prepared by the thin film method will crack during production.

**Figure 10:**
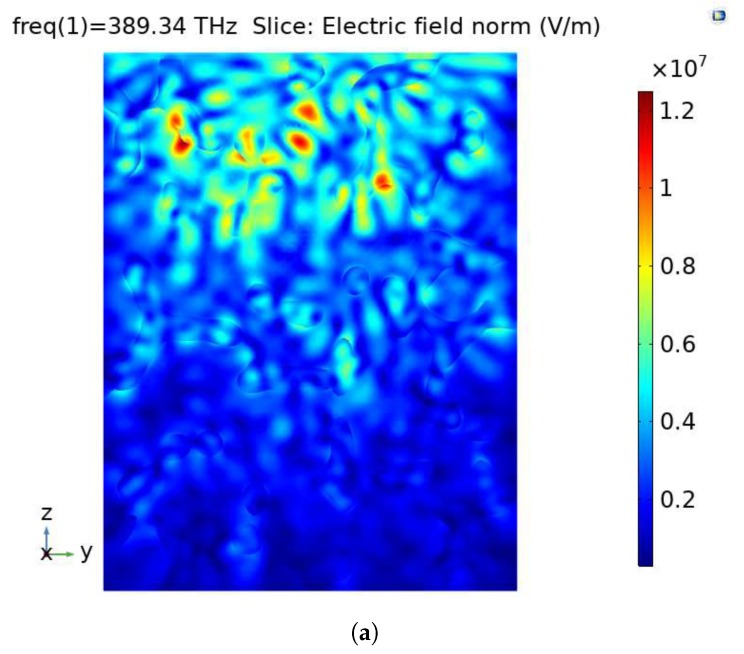

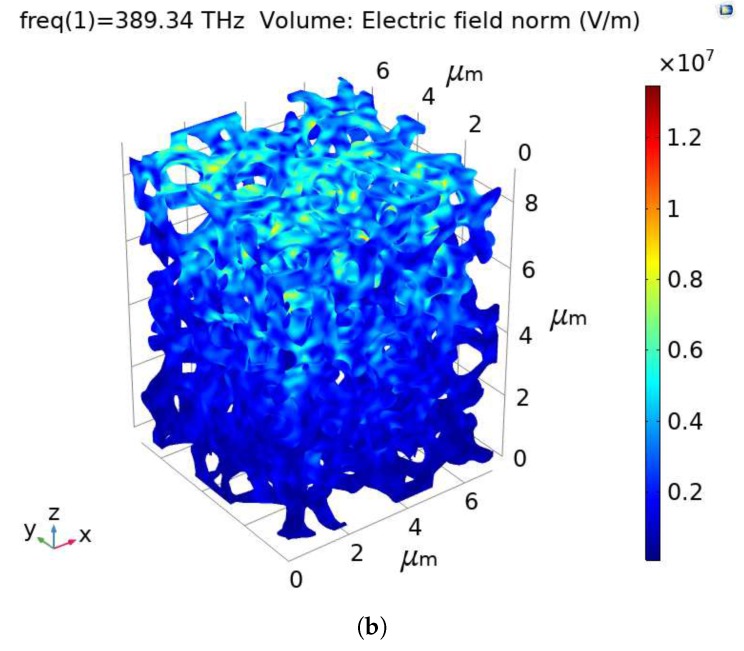
Illustration of the EFN (**a**) in the cutaway view and (**b**) on the surface of the porous mode.

A typical illustration of the EFN inside the model is highlighted in Figure 10. Figure 10a is the cutaway view of the RVE which shows the EFN gradually weakens along the direction of propagation in general. Variations in EFN due to the optical behaviors make the interface between silica and air clearly visible. Figure 10b reflects the distribution of the EFN on the surface of the whole porous model, which also presents a decrease in the EFN along the direction of light propagation.

### 4.2. Influences of the Morphological Parameters

The influences of four morphological parameters on the light transmittance are studied in this section. According to the above analysis, the domain dimension of each model is set as 7×7×9 μm${}^{3}$.

#### 4.2.1. Porosity *p*

**Figure 11:**
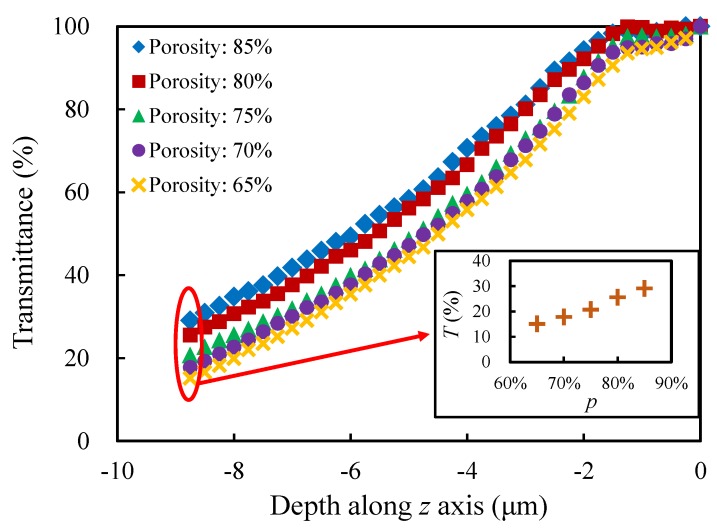
Evolution of the transmittance with the depth along *z* axis for the models with different porosities.

In order to study the influence of the porosity, different sets of models were generated with different porosities (65%, 70%, 75%, 80% and 85%), while the other morphological parameters stay the same, that is,$\left\{C_{V}=0.1;t=1;k=1\right\}$. From Figure 11, one can see that the trend of the light transmittance varying with the depth of light propagation is similar for each set of models with different porosities. As the porosity of the model decreases, the transmittance at the same propagation depth gradually decreases. The small figure reflects the transmittances in the xy-plane of the models with different porosities at the propagation depth z=−8.75 μm, which shows the tendency more clearly. The relative difference between the largest transmittance and the smallest one is over 92%.

#### 4.2.2. Coefficient of Variation $C_{V}$

**Figure 12:**
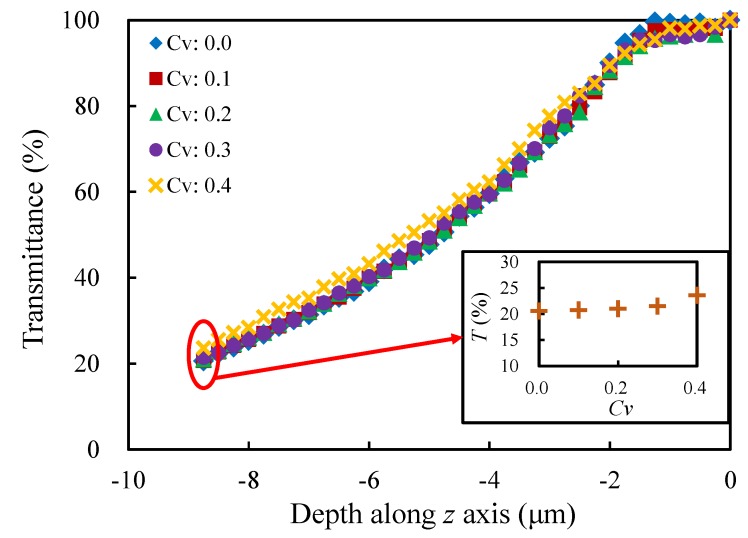
Evolution of the transmittance with the depth along *z* axis for the models with different coefficients of variation.

The cell size distribution is highly controlled by taking different values of coefficient of variation (0.0, 0.1, 0.2, 0.3 and 0.4) so as to study the variation of the light transmittance, while the other morphological parameters are set as $\left\{p=75\%;t=1;k=1\right\}$.Figure 12 presents the influence of the coefficient of variation on the light transmittance. The variation trend of the transmittance is also similar for each set of models with different $C_{v}$s. Furthermore, the influence of the coefficient of variation is smaller on the transmittance of porous models comparing the porosity. The more random the model is, the larger the transmittance is. It can be understood that there are larger pores in the more random structure, and these large pores are more conducive to the propagation of light, whereas the smaller pores appear to have little effect on the propagation. At the propagation depth z=−8.75 μm, the relative difference between the largest and the smallest transmittance is about 14.5%.

#### 4.2.3. Diameter Ratio *t*

**Figure 13:**
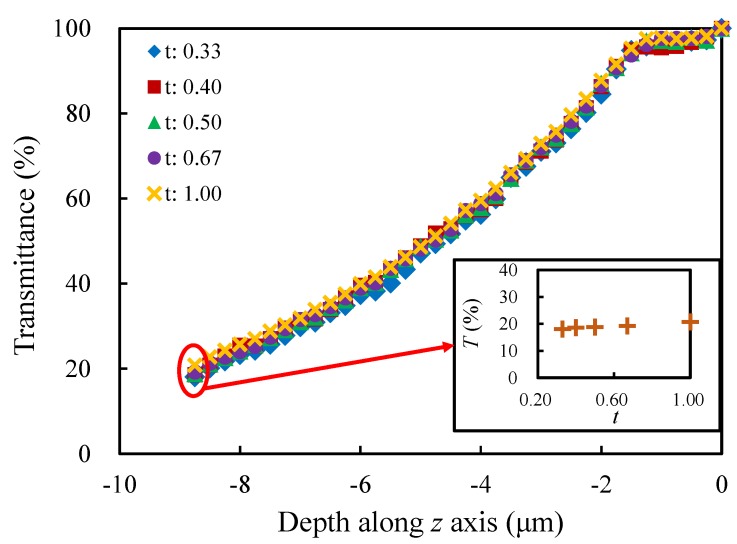
Evolution of the transmittance with the depth along *z* axis for the models with different diameter ratios.

Different diameter ratios (0.33, 0.40, 0.50, 0.67 and 1.00) are taken into account in this section, and the other morphological parameters are the same as those of the sample model. In Figure 13, the curves reflect the similar variation trends for each set of models with different *t*s. In general, the more uniform the struts of porous structure are (the larger *t* is), the larger the transmittance is. The differences may be caused by the relatively complex light propagation when the struts are not uniform (i.e., the two ends are thick and the middle section is thin), which leads to relatively higher probability of absorption, refraction and other losses. At the propagation depth z=−8.75 μm, the relative difference between the largest and the smallest transmittance is about 14.8%.

#### 4.2.4. Normalized Curvature *k*

**Figure 14:**
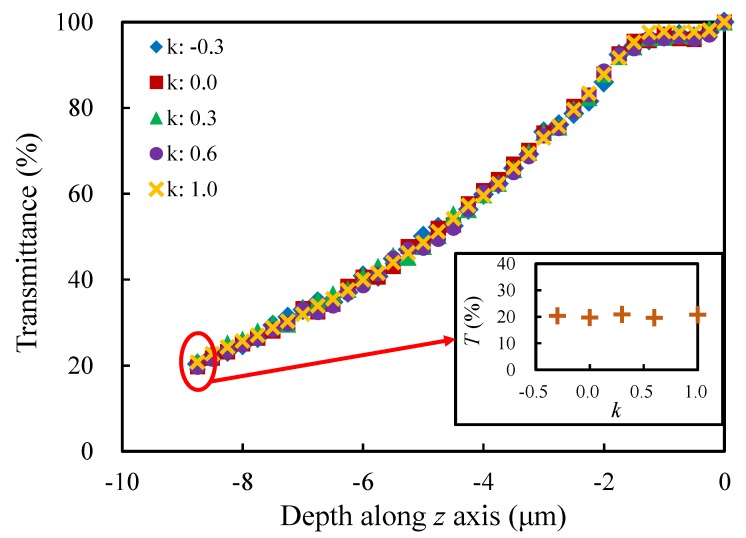
Evolution of the transmittance with the depth along *z* axis for the models with different normalized curvatures.

In this section, the influence of the normalized curvature *k* is studied by controlling *k* to take the values (−0.3, 0.0, 0.3, 0.6 and 1.0). Similarly, the other morphological parameters keep the same values. From Figure 14, except for the same trend as the previous sections, one can see that the normalized curvature has almost no effect on the transmittance. That is, although the cross sections of the porous structural struts have different shapes (concave triangle, positive triangle, convex triangle and circle), since the porous structures are random, the probability of light refracting, reflecting and absorbing on the surface of struts is equal. Hence the light transmittance will not be affected by the shapes of the cross sections.

### 4.3. Influence of the Wavelength λ

**Figure 15:**
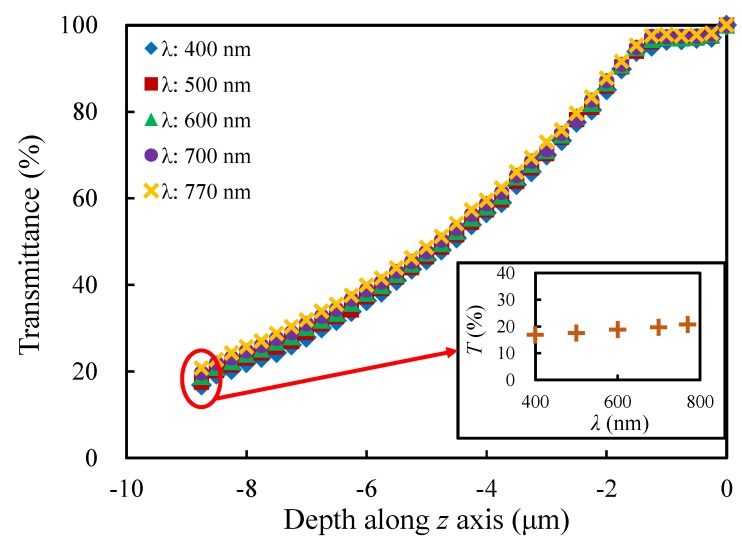
Evolution of the transmittance with the depth along *z* axis for the models under different wavelengths of light.

The light transmittance response of the porous model under different wavelengths of light is the focus of this section. The same porous models as those in Section 4.1 are estimated, and different wavelengths are taken in the visible range (400 nm, 500 nm, 600 nm, 700 nm and 770 nm). Since the effect of the mesh is very small, different global element seed sizes were chosen for different wavelengths, in order to both meet the mesh requirement of COMSOL Multiphysics and minimize the calculation costs. Figure 15 shows the light transmittances of the porous model under different wavelengths of light. It presents that the smaller the wavelength of light is, the smaller the transmittance at the same propagation depth is, which is caused by the greater loss in the propagation process. Moreover, the small figure displays that the relative difference of maximum and minimum transmittance in the visible light range is about 23%.

## 5. Conclusions

In this paper, the light transmittance of porous silica materials used as photocatalyst carriers under visible light is studied by finite element simulation. A series of porous models with different morphologies and structures are generated numerically using the Voronoi-based methodology. The models are controlled to have the same morphology description as those macroporous silicas prepared by sol-gel method. The result shows that the light transmittance decreases quadratically with the increase of propagation depth generally. For the models in this study, when light propagates about 9 μm deep, the intensities are 15–30% of that at the port. The influences of morphological parameters on the light transmittance are studied. Since the refractive index difference between silica and air is very small, in addition to the large influence of the porosity, the coefficient of variation $C_{V}$ and the diameter ratio *t* have relatively small effects, while the normalized curvature *k* has no significant effect. Besides, the wavelength of light λ also has a certain influence on the transmittance. The method introduced in this paper can help to calculate the depth of light propagation inside complex porous bulks, such as porous silica used in photocatalysis, which is difficult to perform experimentally when the propagation depth is very shallow. The study on morphology parameters can provide suggestions for production.

## Figures and Tables

**Table 1:** Illustration of the struts with different diameter ratios.

*t*	0.33	0.5	1
Struts	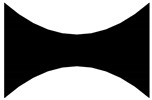	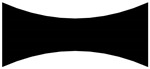	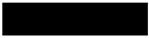

**Table 2:** Illustration of the cross sections with different normalized curvatures.

*k*	−0.5	0	0.5	1
Struts	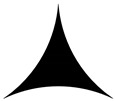	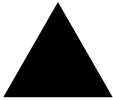	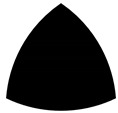	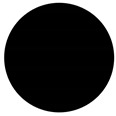

**Table 3:** The variables and their values which are controlled in subsequent sections.

Variable	Range
Porosity *p*	(65%, 70%, 75%, 80%, 85%)
Coefficient of variation $C_{V}$	(0.0, 0.1, 0.2, 0.3, 0.4)
Diameter ratio *t*	(0.33, 0.40, 0.50, 0.67, 1.00)
Normalized curvature *k*	(-0.3, 0.0, 0.3, 0.6, 1.0)
Wavelength λ	(400 nm, 500 nm, 600 nm, 700 nm, 770 nm)
